# Determination of the Phenolic Profile and Antioxidant Properties of *Salvia viridis* L. Shoots: A Comparison of Aqueous and Hydroethanolic Extracts

**DOI:** 10.3390/molecules23061468

**Published:** 2018-06-17

**Authors:** Izabela Grzegorczyk-Karolak, Anna K. Kiss

**Affiliations:** 1 Department of Biology and Pharmaceutical Botany, Medical University of Lodz, Muszynskiego 1, 90-151 Lodz, Poland; 2 Department of Pharmacognosy and Molecular Basis of Phytotherapy, Medical University of Warsaw, Banacha 1, 02-097 Warsaw, Poland; akiss@wum.edu.pl

**Keywords:** antioxidant activity, flavonoids, phenolic acids, phenylethanoids, *Salvia viridis*, UPLC-DAD/ESI-MS analysis

## Abstract

*Salvia viridis* L. is an annual herb used in Mediterranean medicine. The purpose of this study was to determine the polyphenol profile of aqueous (decoction and infusion) and hydroethanolic extracts of aerial parts of field-grown *S.* *viridis* and to evaluate their antioxidant activity. The polyphenol profiling was performed via UPLC-DAD/ESI-MS. Additionally, the total polyphenol content in extracts tested were determined by UV-Vis spectrophotometry using the Folin-Ciocalteu assay. The antioxidant effect was evaluated by the FRAP, DPPH, ABTS, O_2_^•−^ scavenging and TBARS methods. The hydroethanolic extract gave the highest content of total phenolic compounds, followed by the infusion. The UPLC-DAD/ESI-MS analysis of extracts showed a total of 19 phenolic compounds identified as flavonoids (four compounds), phenylethanoids (eight compounds) and phenolic acids (seven compounds). Rosmarinic acid was the predominant phenolic acid, verbascoside was the predominant phenylethanoid, while apigenin glucuronide or methylluteolin glucuronide, depending on the sample, were the predominant flavonoids in the analyzed extracts. The presence of a high polyphenol level indicated a high antioxidant activity of both the infusion and the hydroalcoholic extract. These results indicate that *S. viridis* is a rich resource of phenolic compounds and can be used in dietary applications with the potential to reduce oxidative stress.

## 1. Introduction

*Salvia viridis* L. (*S. horminum*) is an annual herb which grows naturally in the Mediterranean area, Caucasus and Iran. Various cultivars with small inconspicuous flowers, and beautiful pink, violet or white sterile bracts are cultivated as ornamental garden plants. In traditional Turkish medicine, the species has been used as an inflammatory and antiseptic agent in diseases of the throat, gums and eyes [1]. The leaves were also added to fermenting vats to increase the strength and aroma of liquors. In Iran, aerial parts of *S. viridis* has been used for colds and infections, and the seeds for cleaning eyes in inflammation and pain [2,3].

Ulubelen and Brieskorn [4] isolated ursolic, oleanolic and micromeric acids from the aerial parts. Three other triterpenes, i.e., lupeol, lup-(20)29-ene-2α,3β-diol, and olean-13(18)-ene-2β,3β-diol, were later found in the same parts by Ulubelen et al. [5]. Kokkalou and Kapetanidis [6] noted the presence of phenolic acids, including caffeic acid and chlorogenic acid, and flavonoids such as apigenin, luteolin and their 7-glycosides. In recent years, phenylethanoid derivatives have also been found in the aerial parts of *S. viridis variety* Blue Jeans [7].

Plants are an important source of molecules for drug discovery. Phytochemical processing of raw plant materials is required to optimize the concentration of bioactive constituents, with extraction being an important step in the process. The most commonly used extraction techniques include conventional techniques such as infusion, decoction, hot continuous extraction and maceration; however, alternative methods such as ultrasound- or microwave-assisted extraction have also been found to be simple and very effective.

There is a lack of knowledge regarding the detailed phytochemical profile and antioxidant properties of hydrophilic extracts from the aerial parts of *S. viridis*. Therefore, the present study compares the phytochemical profile and activity of various types of extract (decoction, infusion, hydroethanolic extract) of flowering shoots of garden-grown *S. viridis*. The combined use of water and alcoholic solvent may facilitate the extraction of chemicals that are soluble in water and/or organic solvent and is the most suitable method for extracting polyphenolic compounds. Phenol-rich extracts are typically created using a mixture with a 50–80% alcohol content [8,9,10]. Ethanol is safe for human consumption; in addition, ethanolic formulations are more stable than aqueous solutions and are can be subjected to longer storage without losing their value. The present study also evaluates compositions based on infusion and decoction due to their simple preparation, common consumption and low cost. The aim was to obtain high-quality herbal extracts with high yields; this is an important goal if medicinal plants are to be considered as alternatives to synthetic drugs in the prevention and treatment of diseases.

## 2. Results and Discussion

### 2.1. Identification and Characterization of Bioactive Compounds

**Figure 1:**
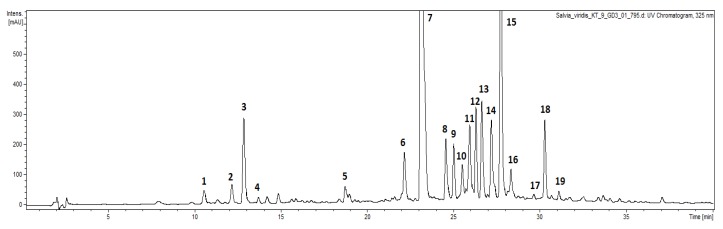
UPLC-UV chromatogram of hydroethanolic extract from aerial parts of *S. viridis.* Peak numbers refer to those used in Table 1.

Numerous *Salvia* species are used in traditional and official medicine as aromatic or phenolic-rich resources. Water and alcohol are good solvents to establish phenolic profile of the plant material. UPLC-DAD-ESI-MS analysis found the different samples to have qualitatively similar content. The full scan negative ionization mode of the *S. viridis* extracts showed a total of nineteen compounds, identified as phenylethanoids (eight compounds), phenolic acids (seven compounds) and flavonoids (four compounds) (Figure 1, Table 1).

#### 2.1.1. Phenolic Acid Derivatives

Peaks **1** (t_r_ = 10.6 min) and **2** (t_r_ = 12.3 min) exhibited a pseudomolecular [M − H]^−^ ion at *m/z* 341. Fragmentation of this ion gave ion at *m/z* 179 due to the loss of 162 amu (hexoside residue), which suggests the presence of a caffeic acid derivative, indicating that compounds **1** and **2** were caffeic acid glucosides [11]; such a structures have been previously found in aerial parts of *S. viridis* [7].

The main pseudomolecular ion obtained from compounds **3** (t_r_ = 12.8 min) and **4** (t_r_ = 13.7 min) was at *m/z* 353 [M − H]^−^. The compounds were identified on the basis of the fragmentation pattern, their elution order and by comparing with available reference standards. According to their substantially different fragmentation behavior [12], the compounds were assigned as 5-*O*-caffeoylquinic acid (chlorogenic acid) (peak **3**) and 4-*O*-caffeoylquinic acid (cryptochlorogenic acid) (peak **4**).

The further compound **5** (t_r_ = 18.7 min) was also characterized as a caffeic acid derivative. It exhibited a pseudomolecular ion [M − H]^−^ at *m/z* 537, and a fragment at *m/z* 341, corresponding to a caffeic acid hexoside. With no further information available, compound **5** was identified as a caffeic acid hexoside derivative. Caffeic acid and its hexosides have previously been reported in *S. viridis* [6] and have commonly been found in other *Salvia* species [13,14].

Compound **11** (t_r_ = 26 min) exhibited a pseudomolecular [M − H]^−^ ion at *m/z* 515. The fragmentation spectrum revealed ion fragments at *m/z* 353 and *m/z* 191 (the deprotonated quinic acid), which confirmed the loss of two caffeic acid units. Dicaffeoylquinic acids are characteristic constituents of the *Asteraceae* family; however, they are very common plant constituents and have also been reported in the *Lamiaceae* family [15]. Our study is the first to report the presence of this compound in the genus *Salvia*.

Peak **15** (t_r_ = 27.7 min) was identified as rosmarinic acid. The fragmentation patterns revealed a pseudomolecular ion [M − H]^−^ at *m*/*z* 359 and fragmentation ions at *m*/*z* 197 and *m*/*z* 161 [M − H]^−^, formed by cleavage of a caffeic acid (−162 amu) and danshensu (−198 amu) moieties. The compound has earlier been detected in aerial parts of *S. viridis* by Rungsimakan and Rowan [7], and it has also been found in roots of the species [16].

#### 2.1.2. Flavonoids

The main pseudomolecular negative ion obtained from compound **6** (t_r_ = 22.1 min) at *m/z* 593 gave a main fragment ion at *m/z* 285. The loss of 308 amu is due to a cleavage of rutinose unit. The ion at *m/z* 285 is attributed to the luteolin aglycone, indicating the compound to be luteolin-*O*-rutinoside [17], a compound previously described in the species by Kokkalou and Kapetanidis [6].

Compound **13** (t_r_ = 26.7 min) was identified as apigenin-*O*-hexuronide by comparing the main fragment ions formed with those described in literature data [18]. Peak 13 showed a fragmentation ion at *m/z* 269, corresponding to apigenin aglycon resulting from a loss of hexuronide [M − H − 176]^−^ moiety from a pseudomolecular ion at *m/z* 445 [M − H]^−^.

Compound **14** (t_r_ = 27.1 min) exhibited a pseudomolecular ion at *m/z* 475 [M − H]^−^ with fragments at *m/z* 299 ([M − 176]^−^) and 285 ([M − 176 − 14]^−^); this was attributed to the successive loss of a hexuronide moiety and a methyl group. These features allowed identifying it as chrysoeriol hexuronide (methylluteolin hexuronide) [19,20]. The glycosides derivative of the methylated flavone was previously been described in other species of *Salvia* [21,22].

Compound **17** (t_r_ = 29.7 min), exhibited a characteristic [M − H − 162] fragment ion at *m/z* 447 and [M − H − 324]^−^ at *m/z* 285 of the pseudomolecular ion at *m/z* 609 [M − H]^−^, implying that it is a dihexoside of luteolin aglycone. Based on MS fragmentation patterns and the published data [23], the compound was identified as a luteolin-*O*-dihexoside. A previous phytochemical study reported the presence of luteolin glycoside and diglycoside in the species [7].

#### 2.1.3. Phenylethanoids

Peak **7** (t_r_ = 23.1 min) and **9** (t_r_ = 25.0 min) exhibited a pseudomolecular ion at *m/z* 623 [M − H]^−^ and fragment ions at *m/z* 461 [M − H − 162]^−^, *m/z* 315 [M − H − 162 − 146]^−^ and *m/z* 135 [M − H − 162 − 146 − 180]^−^ correspond to the initial cleavage of caffeoyl residue and then of rhamnose and hexose units. Both compounds were identified by comparison with the reference standards and literature data as verbascoside and isoverbascoside, respectively [24].

Peak **8** (t_r_ = 24.5 min) showed the same pseudomolecular ion at *m/z* 623 [M − H]^−^ and fragment ion at *m/z* 477 owing to loss of the rhamnose unit (−146 amu) and ions at *m/z* 461, 315 and 135, owing to successive losses of caffeoyl residue (−162 amu), rhamnose and hexose from the main ion. The compound was tentatively identified as an isomer of verbascoside, forsythoside A according to literature data [17,25]. Forsythoside A is rarely found in the Lamiceae family. Although it has been described to occur in *Teucrium* [17] and *Ajuga* plants [26]. This is the first report of its presence in the genus *Salvia*.

Peak **10** (t_r_ = 25.8 min) was tentatively identified as lipedoside A, with a pseudomolecular ion at *m/z* 607 [M − H]^−^. The fragments at *m/z* 461, *m/z* 315 and *m/z* 135 revealed the loss of a coumaroyl (−146 amu), rhamnose and hexose moieties [27]. Again, this is the first report of its presence in the genus *Salvia*.

Peak **12** (t_r_ = 26.3 min) was identified as leucosceptoside. Mitreski et al. [17] report it to have a pseudomolecular ion at *m/z* 637. The fragmentation pattern of this compound gave fragment ions at *m/z* 461 [M − H − 176]^−^, *m/z* 315 [M − H − 176 − 146]^−^ and 135 [M − H − 176 − 146 − 180]^−^. The ion at *m/z* 461 is considered to be the loss of the feruloyl moiety (−176 amu) from the parent ion at *m/z* 637. The ion at *m/z* 315 was produced by the loss of the rhamnose unit from the ion at *m/z* 461, and the ion at *m/z* 135 due to the further loss of hexose. Leucosceptoside has been previously reported from *Salvia viridis* shoots [7].

Compound **16** (t_r_ = 28.4 min) displayed pseudomolecular ion [M − H]^−^ at *m/z* 803. A high abundance of fragments at *m/z* 641 suggested the presence of a caffeoyl residue (−162 amu). Ions at *m/z* 461 and *m/z* 315 indicated the further loss of rhamnose and hexose. However, accurate identification was impossible, and compound 16 was assigned as an unidentified phenylethanoid.

Peak **18** (t_r_ = 30.3 min) was identified as martynoside, giving a pseudomolecular ion at *m/z* 651 [M − H]^−^. The fragmentation pattern of this compound gave ion at *m/z* 475 [M − H − 176]^−^ attributed to the loss of a feruloyl moiety, an ion at *m*/*z* 457, revealing a further loss of water (−18 amu), and ion at *m/z* 505 [M − H − 146]^−^ attributed to the loss of a rhamnosyl group. The ion at *m/z* 329 was produced by the loss of the rhamnose moiety from the ion at *m/z* 475 [28]. The [M − H − feruloyl − rhamnose]^−^ ion of the compound at *m/z* 329 was 14 amu higher than the [M − H − caffeoyl − rhamnose]^−^ ion of verbascoside at *m/z* 315. It revealed that a methoxyl group was added to the phenylethyl moiety. Martynoside has been earlier detected in extracts from the aerial parts of *S. viridis* [7].

For peak **19** (t_R_ = 31.1 min) the LC–MS data also indicated the pseudomolecular ion at *m/z* 651 [M − H]^−^. MS data for this compound was similar to martynoside, suggesting that these two compounds are isomers with similar structures. Based on Abdel-Hady et al. [29], compound **19** was tentatively identified as isomartynoside.

### 2.2. Quantitative Analysis

Biologically-active compounds usually occur in plants at low concentrations, and obtaining them at high yields with minimal changes in composition depends very much on the extraction technique, the yield being influenced by a range of factors including the polarity of the solvent, temperature, pH, extraction time or plant material composition. Therefore, study is required to optimize the extraction method. The present study establishes the quantitative analysis of samples obtained by different extraction procedures. Nineteen phenolic compounds were characterized in the shoots of *S. viridis* based on their retention time, UV spectra and mass fragmentation behavior.

Phenylethanoids were found to be the most abundant group of polyphenols in the studied samples, with the highest content ranging from 1.5 mg/g DW for the decoction to 11.8 mg/g DW for the hydroethanolic sample (Table 2). Among them, verbascoside proved to be the main component of the hydroethanolic extracts and infusions obtained from the aerial part of *S. viridis*, accounting for about 45% and 35% of the total phenol content in the hydroethanolic extracts and infusion, respectively, but only 13% for the decoction. Verbascoside is widely distributed in plants from a variety of families such as the *Lamiaceae*, *Orobanchaceae, Oleaceae* and *Plantaginaceae*. However, only a few reports exist of its occurrence in the genus *Salvia* [7,30]. The compounds possess anti-inflammatory, immunomodulatory and antioxidant properties [31]. Verbascoside has shown good antibacterial activity, particularly against all tested strains of *S. aureus* and *E. coli* [32,33]. Several studies have found it to be beneficial in treating oxidative stress-induced neurodegenerative diseases [34,35]. The compound strongly inhibits the aggregation of amyloid protein [36], and is capable of inhibiting the proliferation of tumor cells, as well as inducing their differentiation and apoptosis [37].

Other compounds based on the phenylethanoids found in *S. viridis* extracts, such as isoverbascoside, forsythoside A, leucosceptoside A and isomartynoside, show equally broad spectra of pharmacological activity [38]. The compounds were detected in both the hydroalcoholic and infusion samples, but in small amounts, ranging from 0.045 to 0.66 mg/g DW (Table 2). Phenyloethanoids accounted for more than half of the total phenolic content of the hydroethanolic extract and 45% of the infusion, but only approximately 20% of the decoction, indicating that of all tested groups, these compounds were the most sensitive to degradation during extraction at high temperature.

**Figure 2:**
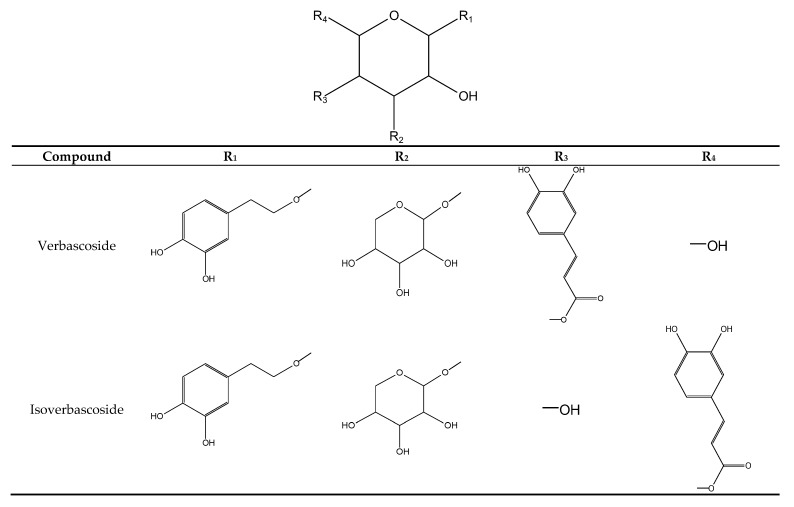

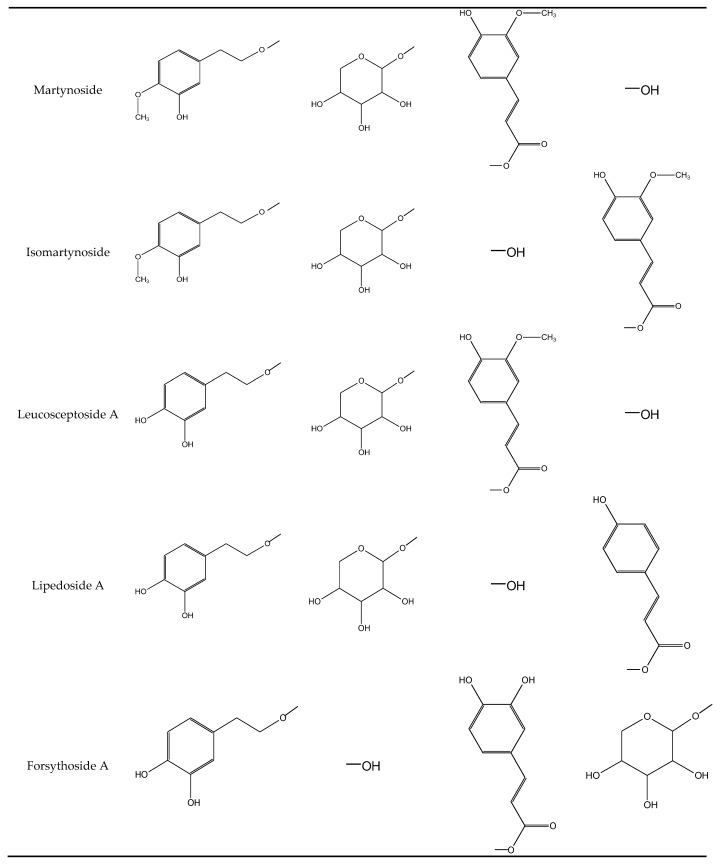
Chemical structures of phenylethanoids in aerial parts of *S. viridis.*

Phenylethanoids have been isolated in large quantities from several Lamiaceae genus of the subfamily Lamioideae, including *Ballota nigra* [39], *Marrubium alysson* [40] or *Sideritis perfoliata* [41]. In contrast, they have been discovered in species of the subfamily Nepetoideae, in which *Salvia* is the largest genus, rarely, for example in *S. officinalis* [42]. Therefore, phenylethanoids have been usually recognized as chemotaxonomic markers between the two subfamilies as defined by Erdtman [42]. Meanwhile, *S. viridis* proved to be one of the exceptional species of the *Salvia* genus, whose predominant metabolites of aerial parts are phenylethanoids (Figure 2).

The predominant group of metabolites for both water samples were the flavonoids, constituting almost 50% of total phenols for infusion and 65% for decoction (Table 2). Similarly, Cvetkovikj et al. [8], noted that the use of ultrasound and 70% ethanol allowed more efficient phenolic acid extraction in sage shoots, but infusion gave the best yield for luteolin derivatives. Our findings confirm the presence of luteolin and apigenin derivatives in all analyzed extracts (Table 2). Apigenin hexuronide was found to be the most abundant flavonoid in the hydroalcoholic extract (4.6 mg/g DW), and methylluteolin-*O*-hexuronide in the decoction (2.4 mg/g DW) (Table 2). The levels of both compounds in the infusion were similar (*cir*. 5 mg/g DW) (Table 2).

The most abundant phenolic acid in all investigated samples was rosmarinic acid (Table 2). According to UPLC analysis, the compound yields ranged from 0.5 mg/g DW (decoction) to 1.3 mg/g DW (infusion and hydroethanolic samples). In all extracts, rosmarinic acid was found to comprise about 70% of total phenolic acid content. It has been found in the aerial parts and/or roots of a number of *Salvia* species [14,43], and has been reported to be the major plant compound responsible for antioxidant activity [44]; the compound helps prevent the cell damage caused by oxidative stress, thereby reducing the risk for atherosclerosis and neurodegenerative diseases. Rosmarinic acid has been found to be a promising drug for cancer prevention and the treatment of various types of human cancers [45]. It also exhibits anti-inflammatory, antiallergic, antibacterial and antiviral effects [44,46,47].

In contrast to our results, rosmarinic acid is often predominant metabolite in the other sage species, and its content in plant aerial parts could be higher than in *S. viridis* shoots. For example, the compound was found as the most abundant phenol in aqueous and alcoholic extracts from aerial part of *S. cadmica* [48]. In that study, RA level was three times greater than in *S. viridis*. On the other hand, similar to our results, methanol extract of *S. cadmica* was found twice times richer in rosmarinic acid than decoction.

In addition to rosmarinic acid, caffeic acid and chlorogenic acid have been previously reported in *S. viridis* shoot extracts [6]. In the present study, chlorogenic acid was identified at concentrations reaching about 300 µg/g DW for hydroethanolic and infusion samples, and 50 µg/g DW for decoction (Table 2). Most caffeic acid was bound to sugars as a hexoside, but was present in its pure form in decoctions (0.05 mg/ g DW; data not shown).

The hydroethanolic and infusion samples demonstrated similar qualitative and quantitative profiles regarding their phenolic acid derivative, phenylethanoid and flavonoid content (about 23–25 mg/g DW). However, some differences were observed for the decoction, especially with regard to total metabolite level (6.6 mg/g DW). The improved yield demonstrated by the hydroethanolic extract may be accounted for by the cavitational effects caused by high intensity ultrasound. Li et al. [49] suggest that ultrasound may reduce the extraction time by increasing the permeability of plant cells, with similar observations being made by Goltz et al. [50] and by Cvetkovikj et al. [8]. In the present study, hot water was also found to be beneficial for obtaining extracts rich in bioactive compounds from *S. viridis* shoots; however, a longer boiling time was found to result in a decrease in extraction efficiency, indicating that some compounds, especially those from the phenylethanoid group, may have undergone degradation. As a result, additional peaks were observed in the sample between minutes 2 and 12 (Figure S1). The compounds were detected as deprotonated ions [M − H]^−^: quinic acid (*m/z* 191) (**1d**), hydroxytyrosol hexoside (*m*/*z* 315 → *m*/*z* 153) (**2d**), caffeic acid hexosides (*m*/*z* 341 → *m*/*z* 281, 179) (**4d** and **5d**), caffeic acid rutosides (*m*/*z* 487 → *m*/*z* 179) (**3d** and **6d**) and caffeic acid (*m*/*z* 179) (**7d**). Moreover, we were able to identified between minutes 17–28 further products of phenylethanoids transformation, nascent due to the oxidation processes: β-hydroxyverbascoside/β-hydroxyisoverbascoside/ β-hydroxyforsythoside A (*m/z* 639 → *m/z* 621, 529, 459, 179) (**8**–**10d**), β-oxoverbascoside syn. β-oxoacteoside (*m/z* 637 → *m/z* 475, 329) (**11d**) and other minor oxidized verbascoside derivatives (*m/z* 621 → *m/z* 458, 487, 179) (**12**–**14d**) (Figure S1). Such products were previously detected in the rhizome of *C. deserticola*, in *Olea europaea* cultured cells, as well as in olive mill wastewater [51,52,53], but are rare and minor compounds in untreated plant materials [54,55]. Noteworthy, flavonoids were stable in the same conditions (Figure S1). The comparison of hydroethanolic extract and the decoction is shown in Supplementary Materials (Figure S1).

Although the low molecular weight degradation products were found in the extract in low amounts ranging from traces to 0.06 mg/g DW, the presence of hydroxy phenylethanoids could influence the measurement of total phenolic compound content in the decoction. These suggestions can be confirmed by comparing the total phenol contents in individual samples determined as the sum of all analyzed compounds with those obtained on the basis of the spectrophotometric method. Folin-Ciocalteu assay found the highest total phenol content to be in the extract prepared with mixture of water and ethanol (105.99 ± 3.03 GAE mg/g) followed by infusion (101.29 ± 1.17 GAE mg/g) and decoction (62.62 ± 1.17 GAE mg/g), with the phenol amount being about 1.6–1.7 times higher in the infusion and hydroethanolic extracts than the decoction. However, when calculated as the sum of the concentrations of all individually-quantified phenolic acid derivatives, phenylethanoids and flavonoids, the total phenolic content was found to be 3.6–3.7 times higher in the infusion and hydroethanolic samples than the decoction. This difference indicates that the decoction contains compounds with –OH groups, as hydroxyphenylethanoids which were not quantified by UPLC analysis.

### 2.3. Antioxidant Properties

The presence of flavonoids, caffeic acid derivatives and phenylethanoids in *S. viridis* samples can enhance their potential antioxidant properties [56]. However, it is well known that due to variety of antioxidant compounds, the activity of a particular extract is dependent on its composition, method of extraction, the polarity of extraction solvent and the employed assay method. An accurate antioxidant analysis requires the use of more than one method to estimate extract properties [57,58]. Therefore, the present study evaluated the antioxidant activity of extracts of *S. viridis* shoots using a number of complementary in vitro assays classified as the SET- or HAT-type methods: the ferric reducing antioxidant power (FRAP), the DPPH, ABTS, O_2_^•−^ radical scavenging assays, and the inhibition of lipid peroxidation test. This approach allows an assessment to be made of the interactions of the extracts with metal ions and both nitrogen- and oxygen-centered free radicals (DPPH, ABTS, and O_2_^•−^) as well as an evaluation of the activity of extract compounds within physiologically-relevant models of lipid oxidation.

**Figure 3:**
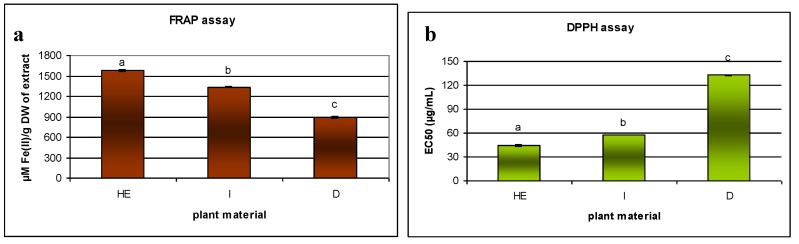

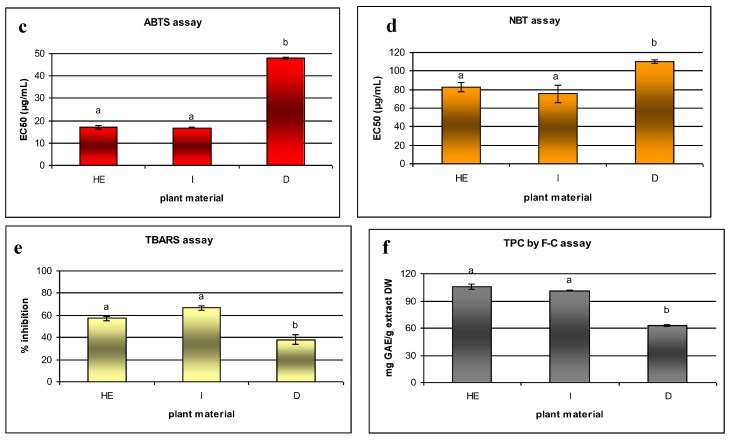
Comparison of antioxidant activity of different aerial parts of *S. viridis* samples (hydroethanolic extract—HE, infusion—I, decoction—D) in FRAP (**a**), DPPH (**b**), ABTS (**c**), NBT (**d**) and TBARS (**e**) assays and TPC (total phenolic content) measured by Folin-Ciocalteu assay (**f**). EC_50_, the concentration of the sample expressed in µg/mL showing 50% of maximal radical scavenging activity. The mean with the same letter do not differ significantly according the one way ANOVA test, followed by the *post-hoc* Tukey’s test for multiple comparisons (*p* ≤ 0.05). The values are means of nine replicates ± standard error.

Data regarding the antioxidant properties of infusion, decoction and the hydroethanolic extracts of *S. viridis* is presented in Figure 3. The obtained data suggest that all extracts possess antioxidant capacity; however, the hydroethanolic and infusion samples showed stronger activity, which were attributed to their higher phenolic compound content. The decoction had the lowest phenol concentrations, and consequently showed the least antioxidant activity.

In this work, the level of antioxidant activity, measured by the scavenging of the radical DPPH, shown by different *S. viridis* samples was as follows: hydroalcoholic extract > infusion > decoction. The scavenging of DPPH radicals for hydroethanolic extract (31.99 ± 0.91 µg/mL) was close to that shown previously by the standard antioxidants: BHT (29.4 µg/mL) or α-tocopherol (23.6 µg/mL) [59]. A previous DPPH analysis of the antioxidant activity of the hydrophilic extract of *S. viridis* shoots showed significantly worse results (570 µg/mL) than those obtained in the present study [60]; however, the extract was obtained using a different procedure based on extraction with water at room temperature, with an unknown duration. The metabolite content of the plant material and hence, its activity, could be affected by a range of other conditions (geographical origin, soil composition, weather) during breeding and timing of collection. Unfortunately, the previous work does not contain phytochemical analysis and it is not possible to analyze any differences in extract composition.

Because of its repeatability and low cost, the DPPH test is the most frequently-used method for assessing antioxidant potential, and has been used to assay the activity of hydrophilic extracts (alcoholic, hydroalcoholic) taken from the above-ground parts of a number of *Salvia* species. The antiradical capacity such known medicinal species as *S. miltiorrhiza* (80% methanolic extract) [61] and *S. officinalis* (methanolic extract) [62] were only slightly greater (EC_50_ value: 17 µg/mL and 23 µg/mL, respectively) than *S. viridis*. Among 16 *Salvia* species of South Africa, the EC_50_ values of their extracts from ranged from about 2 µg/mL, for *Salvia schlechteri*, to more than 100 µg/mL, for *S. radula* and *S. dolomitica* [63]. Meanwhile, five *Salvia* species occurring in Iran were found to show significantly lower activity in DPPH assay than *S. viridis* (EC_50_ value: more than 300 µg/mL) [64].

For the second antiradical assay (ABTS) used in our study, no significant differences were observed between the hydroethanolic and infusion samples. Additionally, as indicated in Figure 3, lower EC_50_ values were obtained by the ABTS assay (16.81 µg/mL for infusion to 47.99 µg/mL for decoction) than by the DPPH one for all samples. Similar results have been obtained for other species, including *Harpagophytum procumbens* [65] or *Rehmannia glutinosa* [66]. Despite the fact that both tests are based on the neutralization of free radicals, there are significant differences between them [67]. For example, DPPH assay is carried out in an aqueous and organic environment (for example alcohol), while the ABTS method requires a buffered aqueous solution. Therefore, some extracts with compounds that are slightly soluble in water, which take part in scavenging the DPPH radical, may not be as active as in the ABTS assay and vice versa. In addition, it has been shown that flavonoids have a relatively high antioxidant activity in ABTS assay; this has been attributed to the formation of flavonoid complexes with ABTS, which also can react with the free radicals, and even have a greater affinity for the radicals than pure flavonoids, reacting more readily with ABTS molecules than flavonoids themselves [68]. Therefore, the ABTS assay may attribute artificially higher antioxidant levels to extracts with high flavonoid levels, and hence other tests should be also used to assess their activity. All samples analyzed in the present study contained flavonoids; however, the greatest amounts of the compounds were found in the infusion, which was more active in ABTS assay than in DPPH test.

FRAP assay exploits the fact that polyphenolic compounds exert their antioxidant activity in the assay through their simple ability to reduce Fe^3+^ to Fe^2+^. Fe and Cu ions are well known as effective pro-oxidant agents in organisms. Polyphenolic compounds have demonstrated the ability to chelate metal ions, thus preventing free radical formation [69]. In addition, the FRAP method is very popular: It is cheap, repeatable, and the reaction proceeds quickly; however, the reaction is not very specific, and it could be argued that the ability to reduce iron has little relationship to the radical quenching process mediated by some antioxidants [57]. In our study, *S. viridis* samples demonstrated FRAP values between 890 and 1575 µM Fe(II)/g DW of extract.

Although the infusion sample demonstrated the highest superoxide anion scavenging activity (NBT) and lipid peroxidation inhibition (TBARS) scores (EC_50_ 75 µg/mL in NBT assay and 66.7% inhibition at extract concentration 100 μg/mL in TBARS method), the results did not differ significantly from those obtained for hydroethanolic extract (respectively, EC_50_ 83 µg/mL and 57.1%). A previous study on the aerial parts of *S. officinalis* gave similar TBARS scores for methanolic extracts (about 55%), but higher ones for acetone extracts (79%) [62]. The TBARS method allows an estimation to be made of the ability of a sample to inhibit malondialdehyde formation resulting from the oxidative degradation of lipids, a process that occurs naturally in the human body or in food products. NBT assay is a method that realistically represents the course of reactions in the human body. O_2_^•−^ radicals, in contrast to synthetic ABTS and DPPH radicals are physiologically important reactive oxygen species involved in various oxidative stress-related reactions, such as the inflammatory, neurodegenerative and aging processes, as well as cancer.

The results obtained in the antioxidant assays were correlated with the quantitative data associated with the phenolic compounds in *S. viridis* samples (Table 3). Several works have reported a strong correlation between polyphenolic compound content and antioxidant activity [59,70]. In all samples tested in the present study, very high correlations were observed between all antioxidant assays and total phenolic compound content, both those designated as the sum of all analyzed compounds (0.888 > |*r*| > 0.997) and those estimated by the Folin-Ciocalteu method (0.908 > |*r*| > 0.999). Correlation studies were applied to identify the main determinants of bioactivity. However, it was found that all tested groups of metabolites are strongly correlated with antioxidant assays. The lowest correlation coefficients were found for the total flavonoid content and FRAP and DPPH assays (respectively, 0.728 and −0.864). However, this may be related to the detailed structure of the specific compounds present in the samples.

It is well known that the antioxidant activity of a compound is greatly dependent on its chemical structure, with higher numbers of free hydroxyls in the molecule usually being associated with greater antioxidant activity. However, molecules with a lower molecular weight can sometimes elicit better reaction kinetics than macromolecules, and could have better access to the active center of the pro-oxidant. In addition, the location of hydroxyl groups and the relationship between them can play a role, as can the type of solvent. Alcohol solutions contain a wider spectrum of compounds than water, including those characterized by antioxidant potential. The variety of structures present in the extract employ a wide range of antioxidative activity mechanisms, which may result in synergistic effects being seen between compounds. On the other hand, alcoholic extracts could also be rich in ballast substances, which can decrease their activities. Although fractionated extraction could allow a purified, standardized product to be produced, the method is too complicated for everyday preparation and use.

Strong or very strong relationships were observed between the tests used to evaluate *S. viridis* antioxidant potential (0.774–0.992; Table 3). Although earlier studies have primarily focused on the roots of *S. viridis* and their diterpenoid constituents, our present findings indicate that the phenol-rich aerial parts of the plant have greater antioxidant potential. They also indicate that this plant material could be used in typical laboratory formulations i.e., hydroethanolic extracts or simple infusions.

## 3. Materials and Methods

### 3.1. Chemicals

Standards of caffeic acid, rosmarinic acid, apigenin-7-*O*-glucoside were purchased from Sigma–Aldrich (Darmstadt, Germany), verbascoside from Phytoplan (Heidelberg, Germany) and isoverbascoside from Roth (Karlsruhe, Germany). Linoleic acid, gallic acid, TBA (2-thiobarbituric acid), TPTZ (2,4,6-tris(2-pyridyl)-s-triazine), ABTS (2,2′azino-bis(3-ethylbenzothiazoline-6-sulphonic acid), potassium persulfate, DPPH (2,2-diphenyl-1-picrylhydrazyl), NBT (Nitroblue tetrazolinum), AAPH (2,2′-azobis(2-amidinopropane)dihydrochloride), Tween 40, PMS (phenazine methosulphate) were purchased from Sigma Chemical Co. (Darmstadt, Germany), and NADH from Roche Diagnostic (Manheim, Germany). Folin–Ciocalteu’s reagent, sodium carbonate, FeCl_3_x6H_2_O and general solvents were obtained from Avantor Performance Materials (Gliwice, Poland). HPLC-grade solvents: acetonitrile, methanol, acetic acid were acquired from J.T. Baker (Arnhem, The Netherlands). 

### 3.2. Plant Material

The aerial parts of *S. viridis* plants were collected from the Garden of the Department of Pharmacognosy at Medical University of Lodz (51°77′ N, 19°49′ E). The seeds (provided by the Garden of Medicinal Plants in Wroclaw) were sown in April, and the plants were harvested at the beginning of August in the flowering phase. The plants were identified by Dr I. Grzegorczyk-Karolak on the basis of the Flora Europaea, and voucher specimens (no. IG/SV1/2015) were deposited in Department of Biology and Pharmaceutical Botany, Medical University of Lodz.

### 3.3. Preparation of Extracts

Lyophilized and powdered plant material (100 mg) was extracted using a UD-20 ultrasonic disintegrator for 15 min with 30 mL of 20:80 (*v*/*v*) water: ethanol solution at 40 °C. The extraction process was then replicated twice more with 10 mL of the same solvent for 15 min. After filtration, the extracts were combined and evaporated to dryness under reduced pressure. In order to explore more suitable forms of obtaining extracts for everyday consumption, the present study investigates two different aqueous extraction procedures:

- Aqueous infusion: Lyophilized and powdered plant material (100 mg) was infused into 30 mL of boiled solvent for 15 min. This process was replicated twice more with 10 mL of the same solvent for 15 min. After filtration, the extracts were combined and evaporated to dryness under reduced pressure.
- Decoction was prepared by boiling of the lyophilized and powdered plant material (100 mg) with the water for 15 min. The extraction process was then replicated twice more with 10 mL of the same solvent for 15 min. After filtration, the extracts were combined and evaporated to dryness under reduced pressure.
  All obtained extracts were stored as dry extracts at 4 °C until analysis.

### 3.4. Qualitative UPLC-DAD-ESI-MS Analysis

The obtained extracts were dissolved in 2 mL of 0.1% HCOOH-MeOH (8:2) and then filtered through a 0.45 μm Chromafil membrane (Machery-Nagel, Duren, Germany). UPLC-DAD-ESI-MS analysis was performed on an UPLC-3000 RS system (Dionex, Germering, Germany) with DAD detection and an AmaZon SL ion trap mass spectrometer with ESI interface (Bruker Daltonik GmbH, Bremen, Germany). Separation was performed on a Zorbax SB C18 column (150 × 2.1 mm, 1.9 μm) (Agilent, Santa Clara, CA, USA) under the general conditions described previously for roots of *S. viridis* [16]. UV spectra were recorded over a range of 200–450 nm and chromatograms were acquired at 325 nm. The LC eluate was introduced directly into the ESI interface without splitting. Compounds were analyzed in negative ion mode. compounds were identified by comparing their retention time, UV–vis and mass spectra with those obtained from standard compounds. Otherwise, peaks were tentatively identified by comparing the obtained information with literature values.

### 3.5. Qualitative Analysis

#### 3.5.1. Total Phenolic Content (TPC)

TPC content was measured using a Folin-Ciocalteu assay according to Grzegorczyk-Karolak et al. [70]. Folin- Ciocalteu reagent (2 mL) diluted ten times was mixed with sample (0.4 mL) and 7.5% Na_2_CO_3_ (1.6 mL). The reaction was performed for 30 min at room temperature. Following this, the absorption was recorded at 765 nm using a Ray Leigh UV-1601 spectrophotometer (Beijing Reyleigh Corp. Beijing, China); identical samples without extract were used as controls. The results, expressed as mg gallic acid equivalent (GAE) per gram dry weight of extract, were obtained against a calibration curve 1 to 400 mg gallic acid.

#### 3.5.2. UPLC Analysis

The samples were dissolved in methanol (2 mL) and centrifuged at 18,000 rpm for three minutes, and the supernatant was analyzed by UPLC. Chromatographic analysis was performed using an Agilent Technologies 1290 Infinity UPLC apparatus equipped with a diode array detection (DAD), a binary solvent delivery pump, vacuum degasser, an autosampler (injection volume 2 µL) according to Grzegorczyk-Karolak et al. [16]. The follow rate was 0.3 mL/min. The detection wavelength was set at 328 nm. When a pure reference standard was not available, the phenolic compounds were quantified according to the calibration curve of another compound from the same phenolic group: flavonoids were quantified according to a apigenin-7-*O*-glucoside curve, phenylethanoids according to verbascoside, and phenolic acid according to caffeic acid. The total content of each different chemical group was calculated by summing the concentrations of the individual quantified compounds. The results were expressed in mg per g DW (dry weight).

### 3.6. Antioxidant Activity

#### 3.6.1. Ferric Reducing Antioxidant Power (FRAP) Assay

The assay was performed according to Grzegorczyk-Karolak et al. [70]. Briefly, 3 mL of fresh prepared FRAP reagent was mixed with 0.3 mL of redistilled water and 0.1 mL of the sample. The samples were incubated for 15 min at 37 °C in darkness. Following this, the absorbance was measured at 595 nm. The antioxidant activity was determined against a standard calibration curve of 0–2000 µM ferrous sulfate. The results were expressed as µM Fe(II)/g of dry extract.

#### 3.6.2. DPPH Assay

The assay was performed according to Grąbkowska et al. [65]. Briefly, 2 mL of analyzed extract at different concentration (2, 20, 50, 100, 200 and 500 µg/mL) was mixed with 2 mL freshly prepared 0.2 mM solution of 1,1-diphenyl-2-picrylhydrazyl radical (DPPH) in methanol. The reaction was performed at room temperature in the dark. The absorption at 517 nm was recorded after 30 min. The antiradical activity was expressed as EC_50_ (µg/mL of dry extract), defined as the concentration of the sample required to reduce the initial DPPH concentration by 50%.

#### 3.6.3. ABTS Assay

The ability to scavenge ABTS was performed as described by Grzegorczyk-Karolak et al. [71]. Briefly, 2 mL samples in different final concentrations (1, 2.5, 5, 10, 25, 50 and 100 µg/mL) were mixed with 2 mL of ABTS stock solution prepared earlier. After 10 min of incubation in the dark, the absorbance of the mixtures was measured in a spectrophotometer at 734 nm. The results were expressed as the EC_50_, i.e., the concentration of the extract able to inhibit 50% of the ABTS radical.

#### 3.6.4. Scavenging of Superoxide Anion Radical (O_2_^•−^)—NBT Assay

The activity was measured based on the reduction of NBT (nitroblue tetrazolinum) according to Nishimiki et al. [72]. Briefly, 1 mL of NBT solution (156 µM NBT in 0.1 M phosphate buffer pH 7.4) was mixed with 1 mL of NADH solution (468 µM in 0.1 M phosphate buffer pH 7.4) and 1 mL of ethanolic extract at different concentrations (1, 5, 10, 25 50 and 100 µg/mL). The reaction was started by adding 0.1 mL of PMS (phenazine methosulphate) solution (60 µM in 0.1 M phosphate buffer pH 7.4). After incubation for five min at room temperature, the absorbance was measured at 560 nm. The activity was expressed as EC_50_ (µg/mL of dry extract), calculated as the concentration of sample demonstrating 50% of maximum absorption.

#### 3.6.5. TBARS Assay

AAPH-induced peroxidation of linoleic acid was determined by the TBARS test according to Marchelak et al. [73]. The method measures the ability of a sample to inhibit the oxidation of linoleic acid. Briefly, 0.3 mL plant extracts at a concentration of 100 µg/mL in the reaction mixture were mixed with 0.7 mL water, 1.4 mL buffer (200 µM, pH 7.4) and 1.4 mL linoleic acid (44 M). Peroxidation was initiated by the addition of 0.2 mL AAPH (53.3 M). The mixture was incubated for three hours at 50 °C. After this time, 1 mL of the reaction solution was mixed with 1 mL 0.05 M HCl, 2 mL Tween 40 and 2 mL 0.67% (*w*/*w*) thiobarbituric acid and heated at 95ºC for 30 min. After cooling, the absorbance of samples was read at 535 nm. The percentage of linoleic acid peroxidation inhibition was calculated using the following equation:

% inhibition= (Abs control − Abs sample − Abs extract) × 100/(Abs control − Abs extract)(1)

where Abs control is the absorbance of ethanol instead of extract.

### 3.7. Statistical Analysis

The estimated values were calculated as means of nine measurements ± standard error (SE). The significance of the treatment effect was determined using Statistica 10.0 software (Statsoft Poland, Krakow, Poland): the results were subjected to one-way ANOVA, followed by the *post hoc* Tukey’s test for multiple comparisons. A 5% probability level was assumed to be significant.

The EC_50_ and correlation coefficients between the antioxidant assay and the total content of phenols obtained by the Folin-Ciocalteu method, as well as total analyzed phenolic content, total phenylethanoid, total flavonoid and total phenolic acid content estimated by UPLC method were calculated using MS-Excel (Microsoft Sp. z o.o. Warsaw, Poland).

## 4. Conclusions

The present results confirm that the shoots of *S. viridis* are highly valuable sources of bioactive phenolic compounds. In total, 19 phenols were identified in the *S. viridis* extracts, some of them for the first time. Both the hydroethanolic (80%) extract obtained by ultrasonic cold extraction, and the infusion obtained by warm extraction with water offer great potential as ingredients in functional food and as preventive agents against diseases caused by oxidative stress. However, further studies are necessary to identify the changes produced by human digestion which could influence the bioavailability and activity of these compounds.

## Figures and Tables

**Table 1:** UPLC-DAD-ESI-MS^n^ data of detected and identified polyphenolic compounds in extracts of aerial parts of *S. viridis.*

	Compounds	Retention Time [min]	UV [nm]	[M − H]^−^	Fragmentation Ions
**1**	6-*O*-caffeoylglucose (I)	10.6	324	341	323, **281**, 251, 221, 179
**2**	6-*O*-caffeoylglucose (II)	12.3	325	341	323, **281**, 251, 221, 179
**3**	5-*O*-caffeoylquinic acid (chlorogenic acid) ^a^	12.8	325	353	**191**
**4**	4-*O*-caffeoylquinic acid (cryptochlorogenic acid)	13.7	325	353	191, **173**, 135
**5**	caffeoyl-hexoside derivative	18.7	325	537	**519**, 341, 281, 179
**6**	luteolin-*O*-rutinoside	22.1	253, 269, 344	593	**285**
**7**	verbascoside ^a^	23.1	330	623	**461**, 315, 135
**8**	forsythoside A	24.5	326	623	**461**, 477, 315, 135
**9**	isoverbascoside ^a^	25.0	326	623	**461**, 315, 135
**10**	lipedoside A	25.8	316	607	**461**, 443, 315, 297, 135
**11**	dicaffeoylquinic acid	26.0	328	515	**353**, 191, 179
**12**	leucosceptoside A	26.3	328	637	**461**, 315, 135
**13**	apigenin-*O*-hexuronide	26.7	267, 332	445	**269**, 149
**14**	methylluteolin-*O*-hexuronide (chrysoeriol hexuronide)	27.1	269, 343	475	**299**, 285, 175
**15**	rosmarinic acid ^a^	27.7	327	359	**197**, 179, **161**
**16**	unidentified phenylethanoid	28.4	328	803	**641**, 461, 443, 315
**17**	luteolin-*O*-dihexoside	29.7	255, 360	609	**447**, 429, **285**
**18**	martynoside	30.3	328	651	505, **475**, 457, 329
**19**	isomartynoside	31.1	328	651	505, **475**, 457, 329, 193

^a^ Identified with authentic standards, **in bold**—the most abundant fragmentation ion.

**Table 2:** Contents of phenolic compounds (mg/g DW) in different aerial parts of *S. viridis* samples (hydroethanolic extract, infusion, decoction).

Compound	Hydroethanolic Extract	Infusion	Decoction
Phenylethanoids			
verbascoside (A)	9.60 ± 0.170a	9.10 ± 0.433a	0.870 ± 0.109b
forsythoside A (A)	0.493 ± 0.005a	0.251 ± 0.011b	trace
isoverbascoside (B)	0.474 ± 0.007b	0.657 ± 0.069a	0.388 ± 0.059b
lipedoside A (A)	0.218 ± 0.005a	0.234 ± 0.001a	0.0883 ± 0.005b
leucosceptoside A (A)	0.524 ± 0.011a	0.512 ± 0.030a	0.0136 ± 0.0002b
unidentified phenylethanoid (A)	0.158 ± 0.006b	0.197 ± 0.007a	0.0433 ± 0.008c
martynoside (A)	0.332 ± 0.009b	0.358 ± 0.002a	0.0445 ± 0.005c
isomartynoside (A)	0.044 ± 0.002a	0.035 ± 0.005a	trace
Total phenylethanoids (TP)	11.843 ± 0.215a	11.344 ± 0.558a	1.448 ± 0.167b
Polyphenolic acids			
6-*O*-caffeoylglucose (I) (C)	0.033 ± 0.001a	0.040 ± 0.0004a	0.011 ± 0.002b
6-*O*-caffeoylglucose (II) (C)	0.066 ± 0.001a	0.047 ± 0.0002b	0.016 ± 0.002c
5-*O*-caffeoylquinic acid (D)	0.354 ± 0.026a	0.313 ± 0.006a	0.054 ± 0.01b
4-*O*-caffeoylquinic acid (D)	0.019 ± 0.0009c	0.026 ± 0.0008b	0.083 ± 0.003a
caffeoyl-hexoside derivative (C)	0.039 ± 0.0007b	0.088 ± 0.001a	0.066 ± 0.017ab
dicaffeoylquinic acid (C)	0.124 ± 0.003a	0.054 ± 0.001b	0.051 ± 0.005b
rosmarinic acid (E)	1.267 ± 0.058a	1.283 ± 0.050a	0.525 ± 0.145b
Total polyphenolic acids (TPA)	1.902 ± 0.091a	1.81 ± 0.07a	0.806 ± 0.18b
Flavonoids			
luteolin-*O*-rutinoside (F)	1.662 ± 0.029a	1.630 ± 0.015a	1.236 ± 0.111b
apigenin-*O*-hexuronide (F)	4.580 ± 0.111b	5.287 ± 0.016a	0.988 ± 0.033c
methylluteolin-*O*-hexuronide (F)	2.661 ± 0.050b	5.163 ± 0.655a	2.406 ± 0.199b
luteolin-*O*-dihexoside (F)	0.143 ± 0.003a	0.0659 ± 0.006b	0.015 ± 0.002c
Total flavonoids (TF)	9.046 ± 0.193b	12.146 ± 0.692a	4.299 ± 0.345c
Total phenolic compound	22.791 ± 0.499a	25.341 ± 1.320a	6.553 ± 0.696b

The mean with the same letter, where “a” corresponded to the highest values, do not differ significantly according the one way ANOVA test, followed by the *post-hoc* Tukey’s test for multiple comparisons (*p* ≤ 0.05). Standard calibration curves: (A) verbascoside—y = 749.68x, *r*^2^ = 0.997; (B) isoverbascoside—y = 542.991x, *r*^2^ = 0.999; (C) caffeic acid—y = 1820.528x, *r*^2^ =0.999); (D) chlorogenic acid y = 970.279, *r*^2^ = 0.999; (E) rosmarinic acid—y = 980.968x, *r*^2^ = 0.999; (F) apigenin glucoside y = 149.724x, *r*^2^ = 0.999.

**Table 3:** Correlation coefficients (*r*) between antioxidant activity values and phenolic contents.

Assay	Antioxidant Activity Method				
	FRAP	DPPH	ABTS	NBT	TBARS
TP	0.952	−0.997	−0.999	−0.970	0.930
TPA	0.932	−0.900	−0.999	−0.983	0.951
TF	0.731	−0.866	−0.922	−0.981	0.998
TPC	0.886	−0.968	−0.992	−0.997	0.979
TPC (by Folin-Ciocalteu)	0.968	−0.999	−0.995	−0.954	0.908
FRAP	-	−0.974	−0.938	−0.849	0.774
DPPH	−0.974	-	0.992	0.947	−0.897
ABTS	−0.938	0.992	-	0.980	−0.946
NBT	−0.849	0.947	0.980	-	−0.992
TBARS	0.774	−0.897	−0.946	−0.992	-

TP-total phenylethanoids, TPA-total polyphenolic acid, TF-total flavonoids, TPC-total phenolic compound.

## Supplementary Materials

The following are available online. Figure S1: The comparison of UPLC-UV chromatogram of decoction and hydroethanolic extract from aerial parts of *S. viridis*, Figure S2: UV spectra and MS spectra in negative ion mode of phenolic compounds from extracts of shoots of *S. viridis*.
