# Supplementary material for: Determination of the Phenolic Profile and Antioxidant Properties of Salvia viridis L. Shoots: A Comparison of Aqueous and Hydroethanolic Extracts
Source: Molecules. 2018 Jun 17;23(6):1468. doi: 10.3390/molecules23061468 (PMC6099398; doi:10.3390/molecules23061468)
Supplement: Supplementary file 1 [file molecules-23-01468-s001.pdf]

Supplementary materials

Figure S1.
The comparison of UPLC-UV chromatogram of decoction and hydroethanolic extract from aerial parts of *S. viridis*

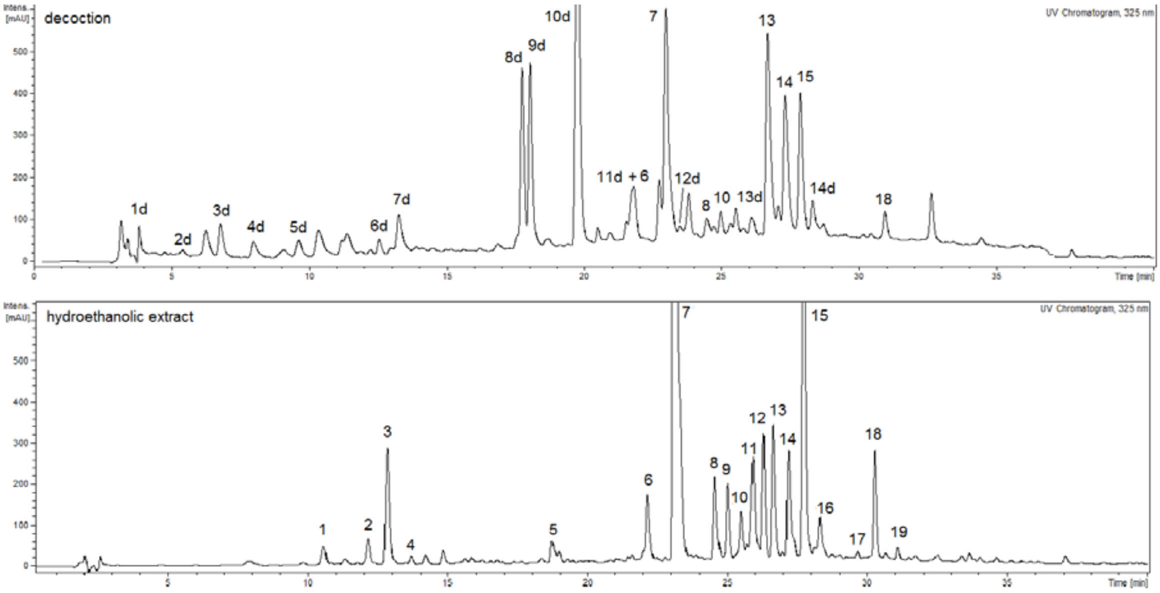

1- 6-*O*-caffeoylglucose, 2- 6-*O*-caffeoylglucose, 3- 5-*O*-caffeoylquinic acid, 4- 4-*O*-caffeoylquinic acid, 5- caffeoyl-hexoside derivative, 6- luteolin-*O*-rutinoside, 7- verbascoside, 8- forsythoside A, 9- isoverbascoside, 10- lipedoside A, 11- dicaffeoylquinic acid, 12- leucosceptoside A, 13- apigenin-*O*-hexuronide, 14- methylluteolin-*O*-hexuronide, 15- rosmarinic acid, 16- unidentified phenylethanoid, 17- luteolin-*O*-dihexoside, 18- martynoside, 19- isomartynoside, 1d- quinic acid, 2d- hydroxytyrosol hexoside, 3d- caffeic acid rutoside, 4d- caffeic acid hexoside, 5d- caffeic acid hexoside, 6d- caffeic acid rutoside, 7d– caffeic acid, 8d- β-hydroxyverbascoside, 9d- β-hydroxyisoverbascoside, 10- β-hydroxyforsythoside A, 11d- β-oxoacteoside, 12-14d - oxidized verbascoside derivatives.

Figure S2.
UV spectra and MS spectra in negative ion mode of phenolic compounds from extracts of shoots of *S. viridis*.

Peak 1

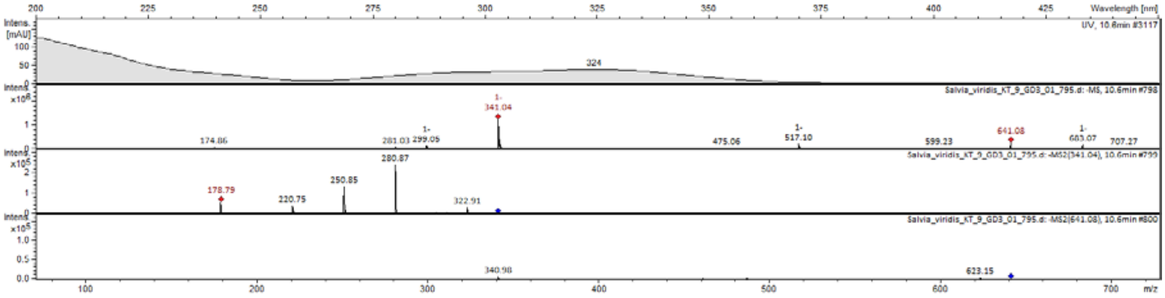

Peak 2

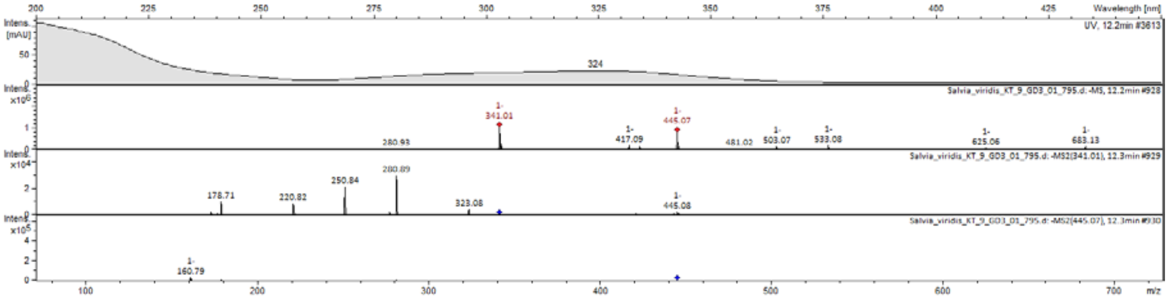

Peak 3

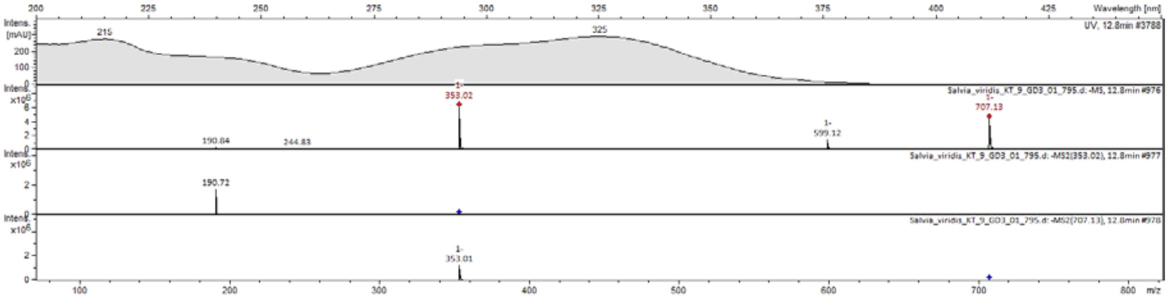

Peak 4

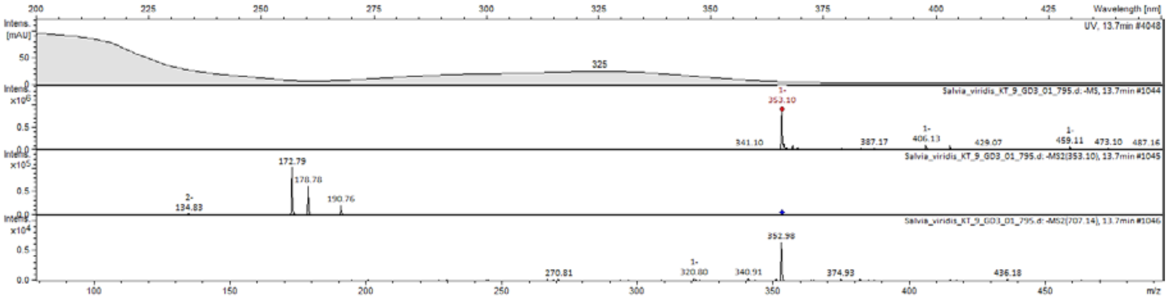

Peak 5

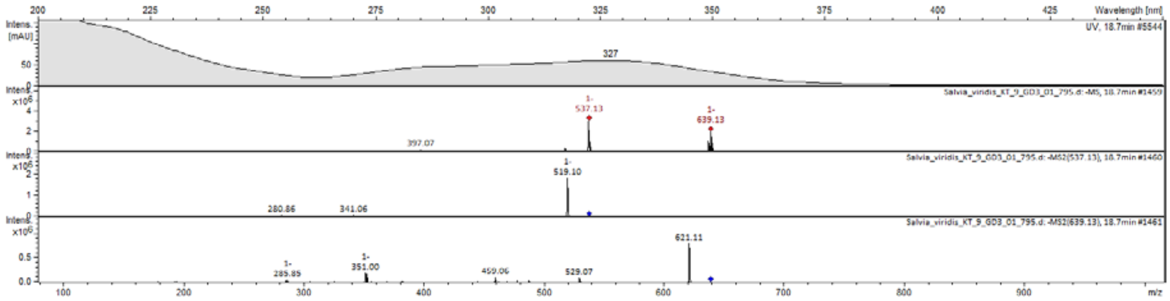

Peak 6

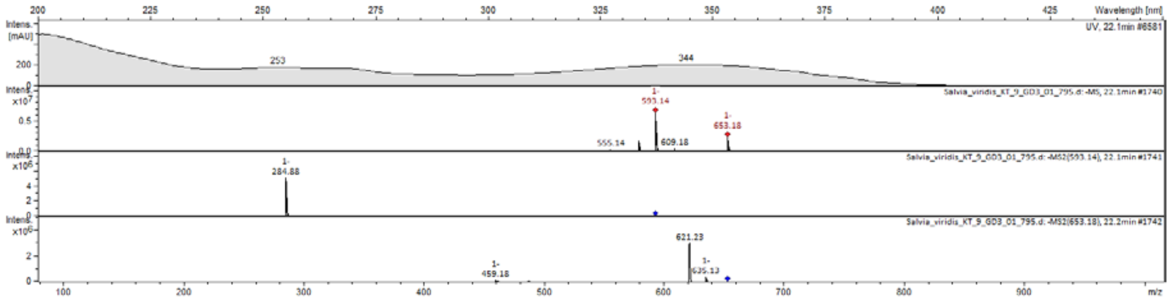

Peak 7

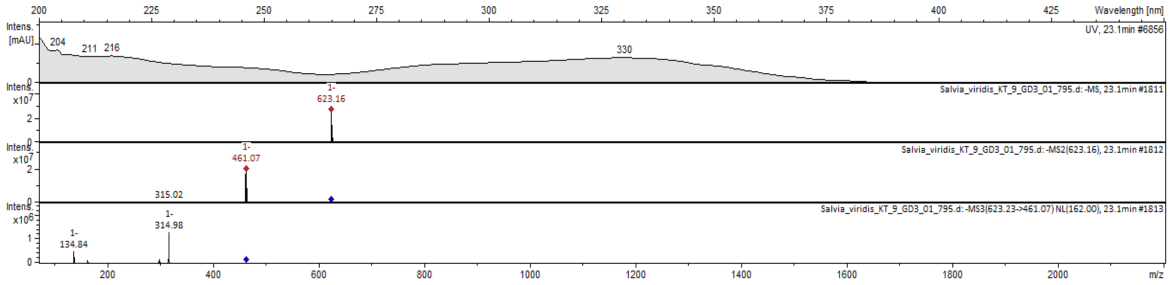

Peak 8

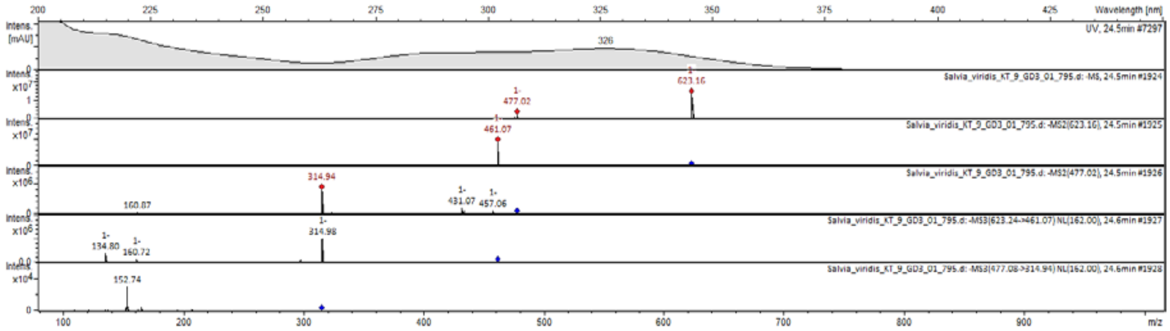

Peak 9

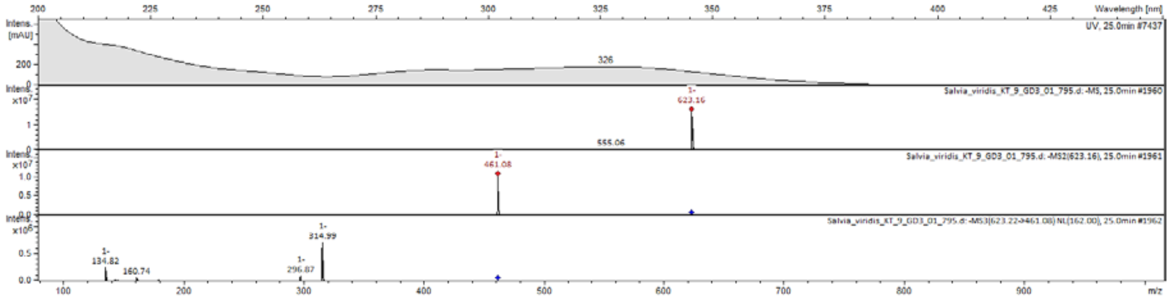

Peak 10

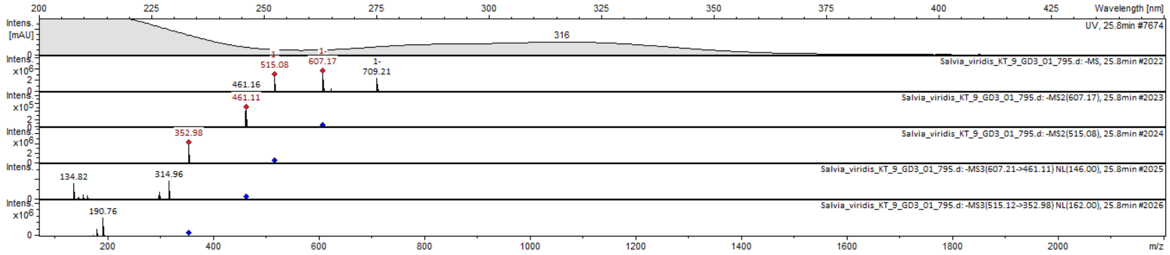

Peak 11

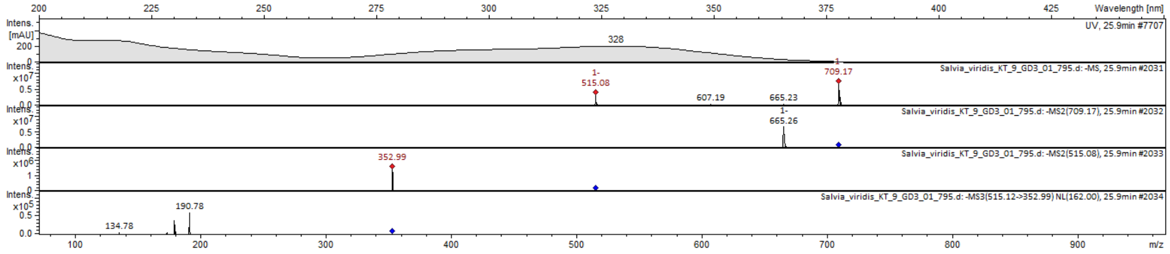

Peak 12

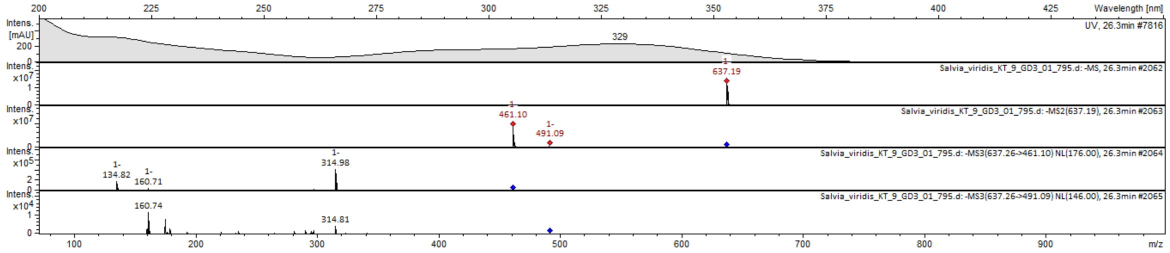

Peak 13

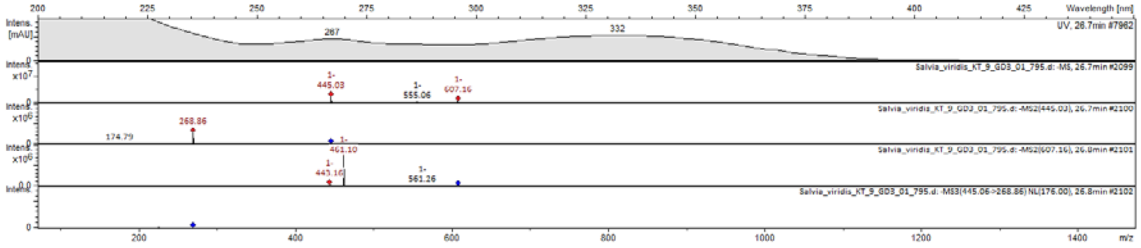

Peak 14

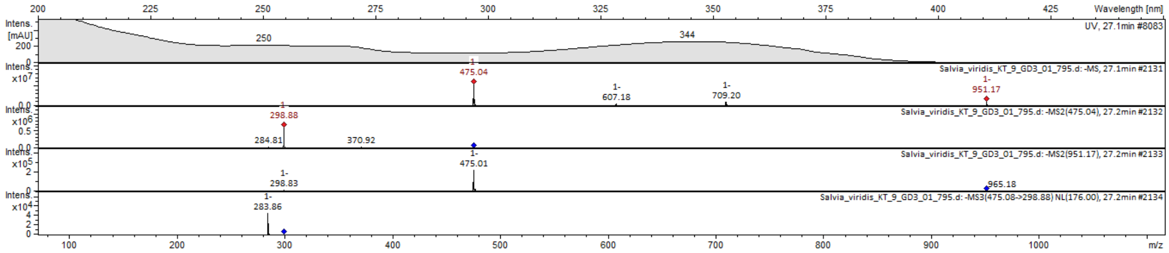

Peak 15

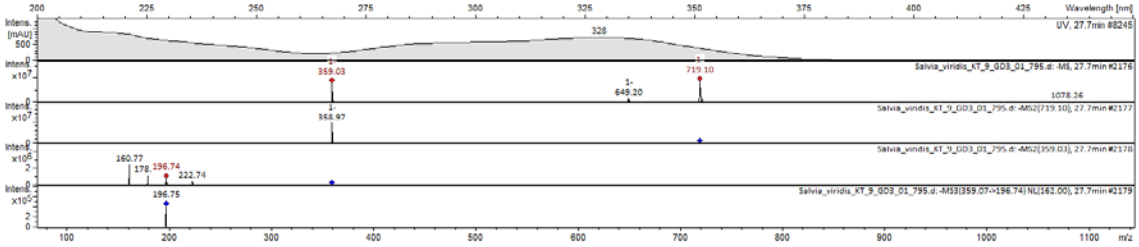

Peak 16

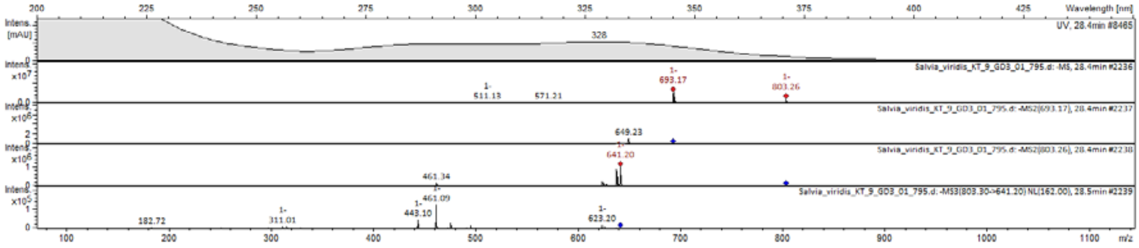

Peak 17

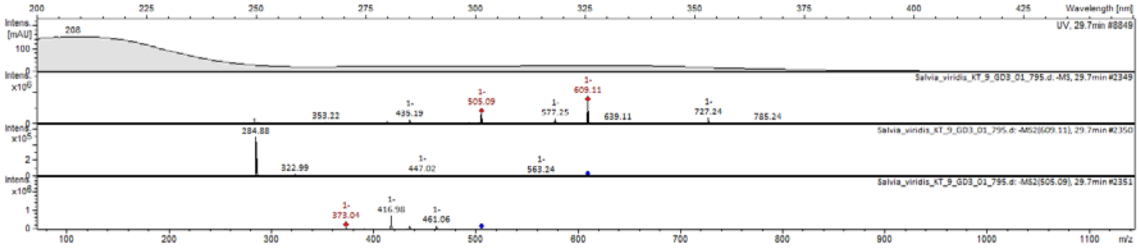

Peak 18

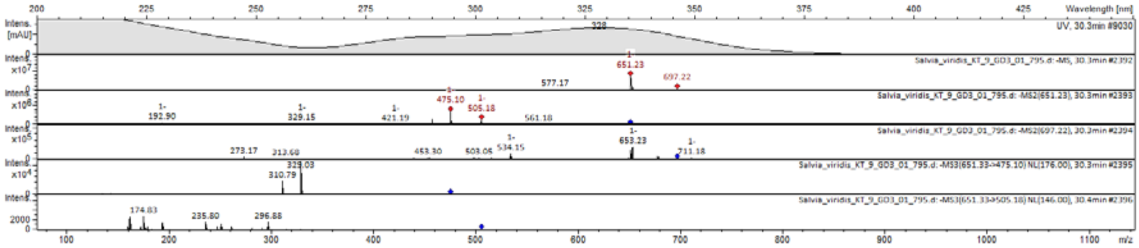

Peak 19

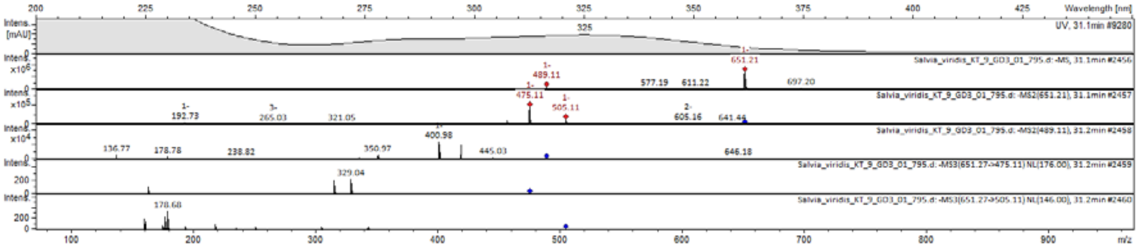
Wavelength [nm]
Intens. [mAU]
UV, 31.1min #9280
325
Salvia_viridis_KT_9_GD3_01_795.d: -MS, 31.1min #2456
1- 651.21
1- 489.11
577.19
611.22
697.20
Salvia_viridis_KT_9_GD3_01_795.d: -MS2(651.21), 31.1min #2457
1- 475.11
1- 505.11
1- 192.73
3- 265.03
321.05
2- 605.16
641.44
Salvia_viridis_KT_9_GD3_01_795.d: -MS2(489.11), 31.2min #2458
400.98
136.77
178.78
238.82
350.97
445.03
646.18
Salvia_viridis_KT_9_GD3_01_795.d: -MS3(651.27->475.11) NL(176.00), 31.2min #2459
329.04
Salvia_viridis_KT_9_GD3_01_795.d: -MS3(651.27->505.11) NL(146.00), 31.2min #2460
178.68
m/z
